# Combined Genome, Transcriptome and Metabolome Analysis in the Diagnosis of Childhood Cerebellar Ataxia

**DOI:** 10.3390/ijms22062990

**Published:** 2021-03-15

**Authors:** Ana Ching-López, Luis Javier Martinez-Gonzalez, Luisa Arrabal, Jorge Sáiz, Ángela Gavilán, Coral Barbas, Jose Antonio Lorente, Susana Roldán, Maria José Sánchez, Purificacion Gutierrez-Ríos

**Affiliations:** 1 CIBER Epidemiology and Public Health (CIBERESP), 28029 Madrid, Spain; ana.ching.easp@juntadeandalucia.es; 2 Andalusian School of Public Health (EASP), 18080 Granada, Spain; 3 GENYO, Centre for Genomics and Oncological Research, Pfizer, University of Granada, Andalusian Regional Government, PTS Granada, 18016 Granada, Spain; luisjavier.martinez@genyo.es (L.J.M.-G.); jose.lorente@genyo.es (J.A.L.); 4 Pediatric Neurology Department, Hospital Virgen de las Nieves, 18014 Granada, Spain; luisillaarrabal@hotmail.com (L.A.); sroldanaparicio@gmail.com (S.R.); 5 Centre for Metabolomics and Bioanalysis (CEMBIO), Chemistry and Biochemistry Department, Pharmacy Faculty, Universidad San Pablo-CEU, 28925 Madrid, Spain; jorge.saizgalindo@ceu.es (J.S.); cbarbas@ceu.es (C.B.); 6 Institute of Biomedicine of Seville (IBIS), 41013 Seville, Spain; gn_angela@yahoo.es; 7 Laboratory of Genetic Identification, Legal Medicine and Toxicology Department, Faculty of Medicine-PTS, University of Granada, 18016 Granada, Spain; 8 Instituto de Investigación Biosanitaria ibs.GRANADA, 18012 Granada, Spain; purificaciongutierrezrios@gmail.com

**Keywords:** cerebellar ataxia, diagnosis, genomics, transcriptomics, metabolomics, hypomyelination, leukodystrophy, *POLR1C*

## Abstract

Ataxia in children is a common clinical sign of numerous neurological disorders consisting of impaired coordination of voluntary muscle movement. Its most common form, cerebellar ataxia, describes a heterogeneous array of neurologic conditions with uncountable causes broadly divided as acquired or genetic. Numerous genetic disorders are associated with chronic progressive ataxia, which complicates clinical management, particularly on the diagnostic stage. Advances in omics technologies enable improvements in clinical practice and research, so we proposed a multi-omics approach to aid in the genetic diagnosis and molecular elucidation of an undiagnosed infantile condition of chronic progressive cerebellar ataxia. Using whole-exome sequencing, RNA-seq, and untargeted metabolomics, we identified three clinically relevant mutations (rs141471029, rs191582628 and rs398124292) and an altered metabolic profile in our patient. Two *POLR1C* diagnostic variants already classified as pathogenic were found, and a diagnosis of hypomyelinating leukodystrophy was achieved. A mutation on the *MMACHC* gene, known to be associated with methylmalonic aciduria and homocystinuria cblC type, was also found. Additionally, preliminary metabolome analysis revealed alterations in our patient’s amino acid, fatty acid and carbohydrate metabolism. Our findings provided a definitive genetic diagnosis reinforcing the association between *POLR1C* mutations and hypomyelinating leukodystrophy and highlighted the relevance of multi-omics approaches to the disease.

## 1. Introduction

Ataxia in children is a common clinical sign of numerous neurological disorders consisting of impaired coordination of voluntary muscle movement that cannot be attributed to muscle weakness [1]. Ataxia usually involves disorders affecting the cerebellum, the vestibular system or peripheral sensory nerves, and these can be acute, intermittent or progressive. Most of the chronic or progressive disorders are consequent to degenerative and metabolic diseases [1,2].

The most frequent form of ataxia—cerebellar ataxia—is caused by damage to, or the dysfunction of, the cerebellum as a result of diverse disease processes. Cerebellar ataxia is a clinically heterogeneous group of disorders that typically manifest with poor coordination, unsteady, staggering gait, limb incoordination (dysmetria), slurred speech (dysarthria), difficulty swallowing (dysphagia), and abnormal eye movements, but it can also exhibit a wide range of phenotypes both in clinical features and in age of onset [1]. This condition is especially devastating for children because they are still developing motor skills [3]. Although the causes of ataxia are countless, they can be broadly divided into two groups: acquired, such as infection or tumor, and hereditary or genetic, such as ataxia telangiectasia [4]. The temporal course of the disorder and the family history are key elements in distinguishing acquired from possible genetic causes [5].

Hereditary cerebellar ataxias are a group of highly heterogeneous disorders, but each usually follows a typical autosomal dominant, autosomal recessive, X-linked or mitochondrial inheritance [5]. Overall, the prevalence of childhood hereditary ataxia in Europe is estimated at approximately 15/100,000 children [6]. Autosomal recessive cerebellar ataxias (ARCAs), with usual onset in childhood, occur more frequently than autosomal dominant cerebellar ataxias (ADCAs), typically having adult onset; which means that recessive causes of ataxia are more common in children. Despite the greater frequency of ARCAs, many of the cases remain not fully characterized and go genetically undiagnosed [7].

There are numerous disease-causing genes currently known for hereditary cerebellar ataxias; however, in spite of the rapid growth of molecular research in childhood presentation of ataxia, diagnostic genetic testing is still difficult owing to the large amount of relatively uncommon subtypes with phenotypic overlap [5]. In addition, cerebellar ataxia is associated with a diverse constellation of neurological features, often overlapping with systemic manifestations, and may also be a major or significant part of the clinical portrait in nearly 300 additional genetic conditions [8,9]. This enormous medical and genetic heterogeneity dramatically complicates clinical management, particularly on the diagnostic stage [10].

Identifying the underlying cause of cerebellar ataxia is a daunting but crucial task [1]. When a diagnosis is accurate and made in a timely manner, a patient has the best opportunity for a positive health outcome [11]. Although few causes of inherited childhood cerebellar ataxia can be treated, making a precise diagnosis is vital for potential therapies, prognostic counseling, and accurate genetic counseling among others [2,12,13]. The diagnostic assessment of a child with cerebellar ataxia is clinically challenging because it requires experience and a methodical approach using detailed clinical evaluation and appropriate investigations [1].

Following detection on a neurological examination of typical clinical signs of ataxia and after the exclusion of non-genetic causes, meticulous and comprehensive investigations to find a genetic cause are required [1]. Unfortunately, in many cases the etiology of ataxia remains uncertain despite a complete workup, and as a result a diagnostic odyssey often begins for these children. This process takes years, imposes heavy economic costs and may not lead to a definitive molecular diagnosis [14].

Advances in omics technologies—genomics, transcriptomics or metabolomics—are promoting a paradigm shift in biomedical science and have ushered in a new era of precision medicine. These technologies can be applied to get a picture of the underlying biology of a disease at a resolution never before seen, and falling costs are now enabling their use in clinical practice and research [15]. Individually, these technologies have contributed to remarkable medical advances that have begun to enter clinical practice. For instance, broadly popular genome and exome sequencing are being progressively incorporated into clinical care, and being used to aid diagnoses, particularly of rare diseases [16,17,18]. While the introduction of these tools in genetic testing has improved the diagnostic yield for genetic ataxias [4], the majority of cases cannot be solved by these technologies alone: a multidisciplinary and combined approach is ultimately needed [15].

Here, we propose exome, transcriptome and metabolome studies to aid in the diagnosis and molecular elucidation of a child presenting with chronic progressive cerebellar ataxia and an undiagnosed condition. Altogether, the results obtained reinforce the association between *POLR1C* mutations and hypomyelinating leukodystrophy [19,20,21,22] as well as the importance of multi-omics approaches to disease.

## 2. Results

### 2.1. Case Report

Our patient was a Spanish girl born to non-consanguineous healthy parents after an uneventful pregnancy and delivery. She had one healthy sister. There were no similar cases in her family, but her father suffered from anxiety and various tics.

When the patient was 2.5 years old, her parents sought medical attention, and a clinical examination revealed tremors, nystagmus, dysmetria and an unsteady gait. Then she subsequently developed progressive ataxia, dysarthria, spasticity and generalized dystonia, requiring the use of a wheelchair at 5 years of age. The patient also exhibited myopia and started wearing glasses at the same age. At age 7, she presented with dysphagia to liquids that worsened over time. At age 8, she experienced a febrile seizure, and treatment with levetiracetam was started, achieving complete control of seizures until the age of 14. Tremors improved after treatment with propranolol. Cognition appeared largely spared and her dentition was normal.

**Figure 1:**
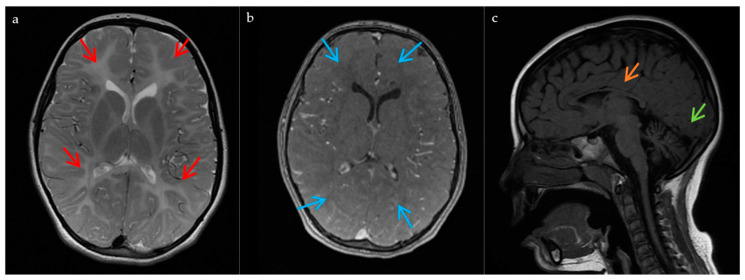
MRI findings in the patient. These images, obtained at the age of 7, show: (**a**) Hypomyelination process with diffuse hyperintensity of the supratentorial and cerebellar white matter on the T2-weighted images (red arrows); (**b**) T1 diffuse isointensity of the supratentorial white matter on the T1 sequences (blue arrows); (**c**) Cerebellar atrophy (green arrow), and a thinned corpus callosum (orange arrow).

A complete metabolic study of blood and urine yielded normal results. Electroencephalography (EEG) recording and nerve conduction velocities revealed no findings of relevance. Visual evoked potentials showed a delayed left P100 wave with bilateral reduced amplitude. Somatosensory evoked potentials were altered in lower limbs. Auditory brainstem response was normal. Electromyography (EMG) confirmed a non-epileptic myoclonus on a deltoid muscle. Magnetic resonance imaging (MRI) of the brain showed diffuse supratentorial and cerebellar white matter hyperintensities on T2-weighted images. Optic radiations were spared. T1 sequence showed diffuse isointensity in the supratentorial white matter suggesting a hypomyelinating process. A thin corpus callosum and cerebellar atrophy (progressive) were also observed (Figure 1). The patient progressively worsened, which accelerated in adolescence, presenting with severe dysphagia (eventually requiring a gastrostomy tube at 14 years old) and more autonomic seizures. She died at 15 from a respiratory tract infection.

The differential diagnosis of known progressive ataxias with leukoencephalopathy included mitochondriopathies, ceroid lipofuscinosis, sialidosis, GM2 gangliosidosis, Niemann Pick type-C disease, and PLA2G6-associated neurodegeneration. The cerebrospinal fluid (CSF) lactate analysis was normal. The muscle biopsy showed a slight deficit of redox complex I and I + III. The Filipin test was normal and *NPC1* gene sequencing was negative, which excluded a diagnosis of Niemann Pick type-C disease. DNA extracted from peripheral blood was sent for sequencing at a hospital. No pathogenic variants were found. Alternatively, high-resolution chromosomal analyses using the whole-genome NimbleGen CGX cytogenetics array (Roche, Basel, Switzerland) by Imegen (Valencia, Spain) revealed a deletion within 16p12.2 (with genomic coordinates chr16:21479157–21647056; GRCh37) of about 0.168 Mb, encompassing genes *METTL9*, *IGSF6*, and *OTOA*. The deletion was classified as being of uncertain clinical significance and was not described before; however, similar deletions in the region had already been described in the general population, suggesting it could be a rare polymorphism. Apart from the *OTOA* gene, known to be associated with an autosomal recessive form of deafness [23], the other genes involved in the deletion had not been previously associated with any medical condition. No other potentially pathogenic losses or gains in other chromosome regions were identified except for known copy number variations (CNVs). In addition, dentatorubral–pallidoluysian atrophy (DRPLA) genetic testing by Lorgen G.P. (Granada, Spain) revealed CAG repeat expansions in the atrophin-1 (*ATN1*) gene within the normal range for the patient, sister and both parents (with 10–18 CAG repeats). Also, mitochondrial DNA analysis of the patient yielded no variants related to mitochondrial diseases apart from one variant (m.1725C>T) not previously described in the literature.

### 2.2. Whole-Exome Analysis

To investigate the etiology and aid in the diagnosis of this unexplained case, next-generation, whole-exome sequencing (WES) was performed on the patient, both parents and her sister. During the process of identifying the causal genes, variants were filtered by frequency <2% and by type (synonymous variants, non-coding intronic, UTR variants). Missense variants, loss of function variants, nonsense and frameshift mutations, splice-site alterations, loss of stop codons, non-synonymous substitutions, and codon insertions and deletions were selected. Variants were then prioritized using values of consequence type, SIFT [24], PolyPhen [25], CADD [26], REVEL [27], MetalLR [28] and MutationAssessor [29]. Sequence analysis revealed (i) two heterozygous variants in the *POLR1C* gene (RNA polymerase I and III subunit C), rs141471029 (c.193A>G; p.Met65Val) in the patient and her mother, and rs191582628 (c.836G>A; p.Arg279Gln) in the patient and her father, and (ii) a heterozygous variant on the *MMACHC* gene (the metabolism of cobalamin associated C), rs398124292 (c.271dup; p.Arg91LysfsTer14), in the patient and her father. The unaffected sibling did not present any of the 3 mutations found in our patient (rs141471029, rs191582628 and rs398124292). These three variants were validated using Sanger sequencing. Both mutations on *POLR1C* induced a missense variation, whereas the one in *MMACHC* resulted in a frameshift variant. Variants were all already registered in the 1000 Genomes Project database, the Single Nucleotide Polymorphism database and the Genome Aggregation Database (gnomAD) [30]. According to the gnomAD database: (i) only 214 out of 251,490 alleles (frequency of allele G = 0.000851) carried the rs141471029 mutation but never in a homozygous state, (ii) only 51 out of 251,432 alleles (frequency of allele A = 0.000203) carried rs191582628 but never in a homozygous state, and (iii) only 290 out of 249,456 alleles (frequency of allele dupA = 0.001163) carried rs398124292. In the European (non-Finnish) population (gnomAD_exome), variant allele frequencies were G = 0.001521 (173/113770), A = 0.000217 (28/129186), and AA = 0.001602 (206/128612) respectively. An in silico pathogenicity assessment performed with PolyPhen-2 [25] and SIFT [24] predicted these variants to be deleterious (pathogenic or likely pathogenic). Both missense variants found at *POLR1C*—a gene that encodes a subunit common to RNA polymerases I (POLR1) and III (POLR3)—affect amino acids that are conserved through species, and have been known to be associated with Treacher Collins syndrome (TCS) [31] and hypomyelinating leukodystrophies [19,20,21,22]. Furthermore, the variant found at *MMACHC* predicts a change of the arginine residue at position 91 to lysine resulting in a frameshift and a premature stop codon. It is also known to be associated with methylmalonic aciduria and homocystinuria cblC type (MAHCC) [32]. The exact function of the protein encoded by this gene is not known, but it is postulated that it could have a role in the binding and intracellular trafficking of cobalamin or vitamin B12 [33].

### 2.3. Transcriptome Analysis

**Figure 2:**
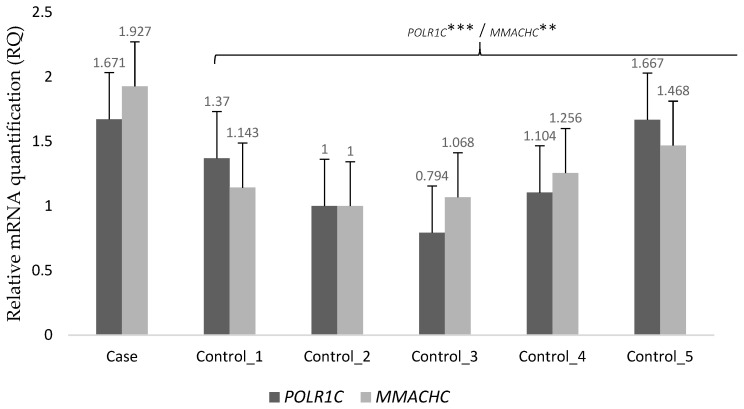
RT–PCR analysis of mRNA expressions of *POLR1C* and *MMACHC* in the patient and the control. mRNA expression was determined by RT–PCR is calculated as a ratio relative to *GAPDH* and expressed relative to the controls. Error bars represent standard deviation (SD). ***, *p* < 0.0001; **, *p* < 0.01.

**Figure 3:**
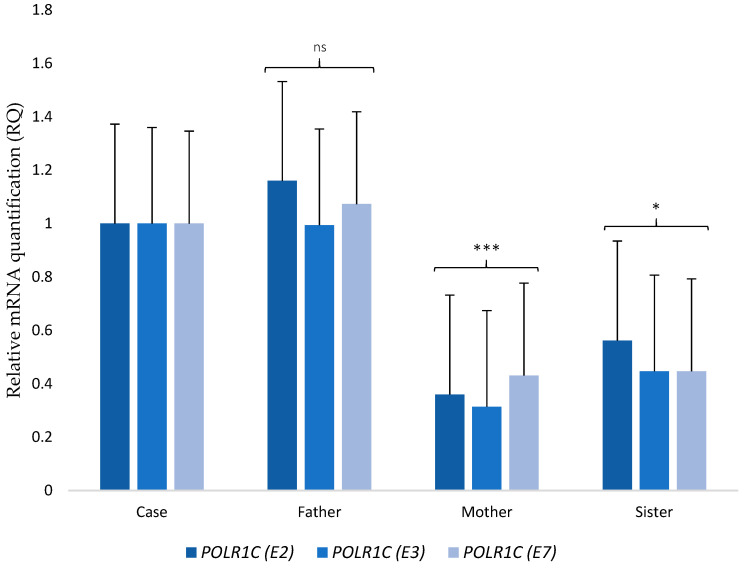
RT–PCR analysis of mRNA expressions of *POLR1C* in the patient and her unaffected family members. mRNA expression determined by RT–PCR is calculated as a ratio relative to *HPRT1* and expressed relative to her parents and sibling. (E2), (E3), and (E7) indicate the different primers used to analyze expression of *POLR1C*. Error bars represent standard deviation (SD). ***, *p* < 0.0001; *, *p* < 0.05; ns, not significant.

To further explore the potential pathogenic role of these mutations, we performed next-generation transcriptome sequencing (RNA-seq) on the patient skin-fibroblast culture, and a human foreskin fibroblast cell culture (HEF line) that served as the control. We compared gene expression as a way to find genes that were differentially expressed between our patient and those of the control. The abnormally expressed genes in our patient can be found in Supplementary Table S1. We found that the candidate genes were within the group of expression outliers—both genes were upregulated. The patient had a 9.5-fold increase in *POLR1C* transcript expression (the logarithm base 2 of the fold change, log_2_FC = 3.244) and a 43-fold increase in *MMACHC* transcript expression (log_2_FC = 5.424) as compared to the control. Increases were validated through quantitative Real-Time Reverse Transcription PCR (RT-PCR) in blood samples from healthy controls. In addition, we set up RT–PCR analyses to study the effect of the variation among the patient and her unaffected family members. Results showed that mRNA levels of *POLR1C* (*p* < 0.0001) and *MMACHC* (*p* < 0.0025) were up-regulated in the patient when compared with the controls (Figure 2). Regarding the differential expression of *POLR1C* in the patient and her family members, both mother (*p* < 0.0001) and sister (*p* < 0.0371) showed significantly lower expression when compared with the patient. On the other hand, the father had higher expression than the mother and sister but similar to that of the patient. Although the gene expression of the father was lower than that of the patient, no statistical differences were found (Figure 3). No malignant isoforms were detected in the patient or any family member studied.

### 2.4. Metabolome Analysis

Using untargeted metabolomic protocols to analyze plasma and urine samples of the patient, a total of 89 and 201 metabolites with known chemical structures were identified, respectively (Supplementary Tables S2 and S3). The levels of 74 plasma metabolites and 187 urine metabolites were increased in the patient compared to the control, while the rest were decreased.

Results from plasma showed that within the phosphatidic acid (PA), phosphatidylcholine (PC), phosphatidylethanolamine (PE) and phosphatidylserine (PS) groups, all metabolites identified, except for PC (14:0) and PS (40:5), were elevated when compared with the control, in a range of log_2_FC 1.5 to 5.6. In addition, within the carnitine pool, all metabolites detected were increased, most of them in a range of log_2_FC 1.5 to 3.1) and one, the 3-Hydroxy-cis-5-tetradecenoylcarnitine, to a higher degree (log_2_FC = 11.8). In the organic acids group, increased levels of lactic acid (log_2_FC = 2.5) and fatty acids (log_2_FC = 1.5–3.4) were found in the patient. With respect to the amino acids profile, the levels of alanine, L-glutamine, asparagine, tryptophan and N-Methyl-L-Proline were increased (log_2_FC = 1.5–2.6), whereas the levels of arginine (log_2_FC = −1.7), cystine (log_2_FC = −2.9) and N6-Methyl-L-lysine (log_2_FC = −16.4) were decreased. Also, two tripeptides were detected with slightly increased levels in the patient as compared with the control. A mild decrease was found in two carbohydrate metabolites, pyranose and furanose (log_2_FC = −1.6). The levels of serotonin (log_2_FC = 1.9), glycerol (log_2_FC = 1.7), urea (log_2_FC = 1.6), and bilirubin (log_2_FC = 1.8) were also increased.

Urine results showed an acylcarnitine profile in the patient where all detected metabolites but one were increased, most in a range of log_2_FC 2.1 to 6.2) except for decadienoylcarnitine with log_2_FC = 9.1, and where the 3-hydroxy−5, 8-tetradecadiencarnitine level was decreased (log_2_FC = −3). PE (22:6) was lower (log_2_FC = −2.6) than in the control. Regarding amino acids metabolism, it should be noted that the level of N-acetyltryptophan was substantially higher (log_2_FC = 9.7), whereas the levels of cystine (log_2_FC = −1.7), lysine (log_2_FC = −1.8), and taurine (log_2_FC = −2.5) were lower in the patient compared with the control. With respect to the organic acids group, most metabolites detected showed levels between log_2_FC = 1.5 and log_2_FC = 5.5 except for the 6-hydroxy-7E,9E-octadecadiene-11,13,15,17-tetraynoic acid (log_2_FC = 7.5) and the 3-hydroxyhippuric acid (log_2_FC = 7.6). The levels of isopentenyladenine-9-N-glucoside (log_2_FC = 9.5), (1R,3S,4S,6R)-6,9-dihydroxyfenchone 6-O-b-D-glucoside (log_2_FC = 9.2), and sulfotrehalose (7,5) were highly increased, whereas the levels of methylsalicylate O-[rhamnosyl-(1->6)-glucoside] (log_2_FC = −1.5), sedoheptulose (log_2_FC = −1.7), 2-methoxyacetaminophen sulfate (log_2_FC = −2.7), and 3-methyl-1-phenyl-3-pentanol (log_2_FC = −3) were decreased in the patient.

## 3. Discussion

We reported on a Spanish patient with *POLR1C* hypomyelinating leukodystrophy. Leukodystrophy due to *POLR1C* mutation is exceptionally rare; so far, only 26 such patients have been reported worldwide [19,20,21,22]. Demographic, genetic, clinical, and radiological characteristics of our patient and those of all published cases are summarized in Table 1, Table 2 and Table 3. In our patient, cerebellar ataxia, tremor and dystonia were the most prominent and severe symptoms.

Clinical and radiological characteristics of our case were compatible with hypomyelinating leukodystrophy due to mutations on the *POLR1C* gene. Hypomyelinating leukodystrophies are a group of heterogeneous, inherited neurodegenerative diseases with overlapping clinical phenotypes and characterized by defects in initial myelin production and formation during development [34]. Mutations in genes encoding subunits of the transcription complex RNA polymerase III (POLR3), namely *POLR3A*, *POLR3B*, *POLR1C*, *POLR3K* and *POLR3GL* might cause a particular type of hypomyelinating leukodystrophies known as Pol III-related leukodystrophies [19,35,36,37,38,39]. POL3 is responsible for the transcription of various non-coding RNAs which have relevant functions in translation and other biological processes. Dysregulation of POL3 transcribed genes is associated with a variety of diseases [40]. Recently, Kraoua et al. concluded that imaging findings were present in the majority of Pol III-related leukodystrophies and could serve a useful role in the clinical setting to distinguish them among other hypomyelinating disorders and so guide the molecular diagnostic workup [20]. Advances in molecular genetics have enabled clinicians to identify more cases having a genetic cause; however, research in the field has historically suffered from a scarcity of medical and scientific knowledge, and all those affected face similar challenges in their search for diagnosis, appropriate treatment and medical care [41]. The management of childhood cerebellar ataxia, a rare condition with high degree of medical and genetic heterogeneity, warrants a broad, multidisciplinary and interdisciplinary approach that includes precision medicine [15,42]. Thus, we used a multi-omics-based approach to diagnose our patient and help advance our understanding of the disease biology.

WES analysis in our patient confirmed two variants in *POLR1C*. *POLR1C* mutations are believed to reduce the transcription of tRNAs or other small noncoding RNAs that are central to the synthesis of proteins crucial for myelin development in the central nervous system [19]; however, the molecular mechanisms underlying *POLR1C* variants causing hypomyelination remain largely unknown [43]. Although it seems clear that mutations in Pol III subunits can lead to defects in the enzyme, the precise consequences of this are difficult to ascertain [40]. To date, 29 pathogenic variants in *POLR1C* have been described [22]. WES also revealed a mutation in *MMACHC* known to be associated with MAHCC, the most common genetic defect of cobalamin metabolism [32]. To date, 55 mutations have been identified. The mutation in our patient (p.Arg91LysfsTer14) is the most prevalent worldwide [44,45]. Transcriptome analysis showed increased expression of *POLR1C* and *MMACHC* in our patient. We hypothesized that there could be a compensation mechanism in place that increases rather than decreases mRNA levels due to the need to maintain the protein at adequate levels. The appearance of a point mutation, such as the ones present in our case, does not necessarily mean a decrease in the quantity of mRNA in the exon. In contrast, if the protein produced is not functional or is truncated, it is reasonable for an increase in its expression to occur in order to compensate for the levels of such protein. This upregulation mechanism has been previously described [46,47]; however, its applicability to this particular case should be further investigated. Transcriptomics analysis of the differential expression of *POLR1C* in the patient and her father also revealed that there was no statistically significant difference in spite of the father’s only presenting one mutation in the *POLR1C* gene and not two as the patient did. The upregulation mechanism hypothesis might also explain why *POLR1C* expression in the father was elevated; nonetheless, since the mutation in the father was not accompanied by any other on that gene, as in the case of the patient, the levels of the protein encoded by *POLR1C* in the father could be sufficient for normal function. Although the mother also shared a *POLR1C* variant with the patient, she did not show an elevated expression of *POLR1C*, whereas the father did. This could be explained by the different clinical significance of both variants, as annotated in ClinVar (NCBI-NIH) [48]. The *POLR1C* variant in the father, rs191582628, is classified as pathogenic or likely pathogenic whereas the variant present in the mother, rs141471029, showed conflicting interpretations of pathogenicity. In addition, an untargeted metabolomic approach to a preliminary examination of plausible functional effects of mentioned mutations, which could also provide further information for finding potential diagnostic biomarkers, revealed a different metabolic profile in our patient compared to the healthy control. The main alterations were found in the metabolism of amino acids, fatty acids and carbohydrates. These results might be relevant since the nervous system, in particular the cerebellum with its high metabolic demand, is highly vulnerable to metabolic perturbations. Hence, many disorders of intermediary metabolism will preferentially target the cerebellum, with ataxia as part of the neurological symptom complex [49]. The *POLR1C* gene encodes for the RNA polymerase I and III subunit C, and the variants found have already been associated with TCS [31] and hypomyelinating leukodystrophy [19,20,21,22]. Among the pathways related to this gene are the RNA polymerase I Promoter Escape, with a total of 49 genes involved, and ATP/ITP metabolism, with a total of 85 genes involved (www.genecards.org). Genetic Ontology (GO) annotations related to this gene include protein dimerization activity and RNA polymerase II activity. Both the pathways and the activities have implications at the metabolic level [50]. In this work we shared the complete, raw and unbiased list of metabolites that were altered in our patient with respect to the control, nonetheless, to define the metabolic implications in the origin or development of the disease caused by the variants of the *POLR1C* gene, functional analyses of the affected pathways are essential.

Compared to studies of single-omics-types, multi-omics provides the opportunity to draw a comprehensive picture of a disease [51]. In our case, a genetic diagnosis was reached, but an integrative approach and further molecular and functional studies are required to achieve a systemic understanding of our patient’s phenotype.

## 4. Materials and Methods

### 4.1. Consent and Approval

Informed consent was obtained from all adult participants, and parental permission and child assent were obtained for child participants. The project was approved by the Biomedical Research Ethics Committee of the Andalusian Public Health System in Granada, Spain on 29 October 2015, (project identification code AP163052016). Data recording, sample collection and all in vitro experiments were conducted in accordance with ethical guidelines following the Nuremberg Code, Belmont Report, and the Declaration of Helsinki.

### 4.2. WES for Candidate Gene Identification

A WES analysis was performed for the patient and all available family members. Genomic DNA was extracted from peripheral blood leukocytes using the QIAamp DNA Blood Mini Kit (Qiagen, Hilden, Germany) and the QIAcube instrument (Qiagen, Hilden, Germany) according to the manufacturer’s instructions, and DNA was sheared to 180–220 bp fragments with Covaris E220 Focused-ultrasonicator (Covaris, Woburn, MA, USA). Exome capture and library preparation were performed using 500 ng of sheared original DNA and an in-solution hybridization technology for target enrichment, Roche NimbleGen SeqCap EZ MedExome (Roche Sequencing Solutions, Pleasanton, CA, USA), following the HyperCap Workflow v2.0 (Roche Sequencing Solutions, Pleasanton, CA, USA). The MedExome design enables the enrichment of medically relevant regions of the human exome. Exome libraries were sequenced on the NextSeq 500 system (Illumina, San Diego, CA, USA) using the highest output mode and paired-end 150 bp read lengths. Reads were aligned to the human reference genome GRCh38 with BWA v0.7.9a [52], SAMtools v1 (https://github.com/samtools/samtools, accessed on 15 March 2021) [53], Picard v2 (http://broadinstitute.github.io/picard, accessed on 15 March 2021) and Qualimap 2 [54], and variant calling and filtering was then performed using both the Genome Analysis Toolkit (GATK) v4 [55,56] and the suggested SAMtools mpileup pipeline [53,57]. Variant annotation and prioritization was completed following standard guidelines and the ANNOVAR tool [58,59], and using Ensembl (https://www.ensembl.org/index.html, accessed on 15 March 2021) [60] as the primary variant annotation database and PolyPhen-2 [25] and SIFT [24] as the in silico tools for evaluating the variants’ pathogenicity. In addition, all variants that produced a change in nucleotide or a change in frameshift were reviewed and reannotated by in silico analysis on the functional and structural impact of the SNPs using Ensembl Variant Effect Predictor (VEP) [61], ClinVar (NCBI–NIH) [48] as well as tools from DAVID Bioinformatics Resources 6.8 (https://david.ncifcrf.gov/, accessed on 15 March 2021) [62,63] and Ingenuity Pathway Analysis (IPA). Integrative Genomics Viewer v2.3 (IGV) (https://software.broadinstitute.org/software/igv/, accessed on 15 March 2021) [64,65] was used to support data visualization and exploration.

### 4.3. RNA-Seq for Gene Expression Analysis

RNA-seq analysis was performed for the patient and a control sample. The patient’s fibroblasts were retrieved from a skin biopsy and the control fibroblasts were retrieved from the HEF line (human foreskin fibroblast cell culture). The fibroblast culture was maintained in DMEM high glucose medium (Thermo Fisher Scientific, Waltham, MA, USA) supplemented with 10% Fetal Bovine Serum, Glutamine. RNA was extracted from primary fibroblasts using the RNeasy Mini Kit (Qiagen, Hilden, Germany) according to the manufacturer’s instructions. Concentration and quality of extracted RNA were measured using the Qubit 4 Fluorometer (Thermo Fisher Scientific, Waltham, MA, USA) and the 2100 Bioanalyzer Instrument (Agilent Technologies, Santa Clara, CA, USA). Libraries from mRNA were prepared using 1 μg of RNA starting material and the TruSeq Stranded mRNA Library Prep Kit (Illumina, San Diego, CA, USA) according to the manufacturer’s protocol. mRNA libraries were sequenced on the NextSeq 500 system (Illumina, San Diego, CA, USA) using the highest output mode and paired-end 75 bp read lengths. RNA-Seq data were aligned to the human reference genome GRCh38 by RSEM v1 (https://deweylab.github.io/RSEM/, accessed on 15 March 2021) [66]. Quantification of sequence alignments was performed at the transcript level using RSEM [66] and differential gene expression was measured using DESeq (https://www.bioconductor.org/packages//2.10/bioc/html/DESeq.html, accessed on 15 March 2021) [67].

### 4.4. Validation of Next-Generation Sequencing (NGS) Candidate Variants

A small number of identified variants were validated in house through amplification and Sanger sequencing with the BigDye Terminator v3.1 Cycle Sequencing Kit (Thermo Fisher Scientific, Waltham, MA, USA) using the capillary electrophoresis instrument ABI PRISM 3130 Genetic Analyzer (Applied Biosystems, Foster City, CA, USA). Sequences were analyzed using SeqScape Software v2.7.

Transcriptomic analyses were validated using quantitative PCR (qPCR) and specific primers designed for the study with Primer3 software [68,69] and RNA extracted from blood samples. Control blood samples came from unaffected family members (parents and sibling) and from healthy volunteers matched for weight, sex and age with the patient. RNA from the patient, controls and unaffected family members was reverse-transcribed into cDNA using the SuperScript VILO cDNA Synthesis Kit (Thermo Fisher Scientific, Waltham, MA, USA), and then used for qPCR using a QuantStudio 6 Flex Real-Time PCR System (Applied Biosystems, Foster City, CA, USA) with iTaq Universal SYBR Green Supermix (Bio-Rad, Hercules, CA, USA) according to the manufacturer’s protocol. Quantification of the fold change in gene expression was determined by relative quantification (RQ; 2^−ΔΔCT^) using the *GAPDH* and *HPRT1* genes as a reference. In addition, transcript isoform identification was performed in the patient and family members through amplification and Sanger sequencing with the BigDye Terminator v3.1 Cycle Sequencing Kit (Thermo Fisher Scientific, Waltham, MA, USA) using the ABI PRISM 3130 Genetic Analyzer (Applied Biosystems, Foster City, CA, USA). Statistical analyses were made with GraphPad Prism version 5.0 for Windows (GraphPad Software, La Jolla, CA, USA, www.graphpad.com, accessed on 1 February 2020). For qPCR analysis, unpaired t-tests were performed to compare the means of the two groups. Values of ΔΔCt out of mean ± SD were considered.

### 4.5. Functional Validation through Metabolomics

#### 4.5.1. Metabolite Extraction and Mass Spectrometry Analysis

Metabolite extraction was performed in plasma and urine samples from the patient and a healthy control, matched for age, sex and weight, according to standard protocols with some modifications. The detailed descriptions of the protocols can be found in the Supplementary Material. Liquid chromatography–mass spectrometry (LC–MS) analyses were performed using a UHPLC system (Agilent 1290 Infinity LC System, Waldbronn, Germany) coupled to a LC-QTOF-MS (6520) analyzer (Agilent Technologies, Santa Clara, CA, USA). Gas chromatography–mass spectrometry (GC–MS) analyses were performed using a 7890A from Agilent Technologies (Waldbronn, Germany) coupled to an inert mass spectrometer with triple-Axis detector 5975C (Agilent Technologies, Santa Clara, CA, USA). Capillary electrophoresis–mass spectrometry (CE–MS) analyses were performed on a 7100 CE system coupled to a 6224 accurate mass TOF MS (Agilent Technologies, Santa Clara, CA, USA), equipped with an electrospray ionization (ESI) source.

#### 4.5.2. Data Treatment

LC–MS and CE–MS data were cleaned of background noise and unrelated ions by the Molecular Feature Extraction (MFE) tool in MassHunter Profinder software B.08.00 (Agilent Technologies, Santa Clara, CA, USA). Each compound was described by mass, migration time (MT) (CE–MS) or retention time (RT) (LC–MS), and abundance. Data were filtered based on their quality assurance. Spectral deconvolution with MassHunter Unknowns Analysis software version B.08.00 (Agilent Technologies, Santa Clara, CA, USA) was used to extract the data acquired by GC–MS analysis. Agilent MassHunter Quantitative Analysis software version B.08.00 was used for the assignment of the target and qualifiers ions, and peak area integration. Prior to the statistical analysis, sample areas were normalized by the internal standard abundance. Data were filtered by the coefficient of signal variation (CV) in QCs. The features or metabolites from the two samples were compared by their absolutes differences in abundance, and that was expressed as the logarithm base 2 of the fold change (log_2_FC). Those features or metabolites having a log_2_FC ≥ 1.5 were considered significant and were selected.

#### 4.5.3. Metabolite Identification

Briefly, compound identification from LC–MS and CE–MS data was performed comparing accurate *m/z* outcome measurements against leading online MS databases (METLIN, KEGG, HMDB and LIPID MAPS) using the CEU Mass Mediator tool. The identification of *m/z* was performed considering each of the possible adducts as described for MFE. Compound identification from GC–MS data was performed with the Fiehn library version 2008 and the CEMBIO in-house spectral library, and always by comparing both RT and spectra extracted during the deconvolution against each compound included in the library.

Additional information on the untargeted metabolomics methodology is provided in the Supplementary Material.

## 5. Conclusions

Our findings add evidence to the genetic spectrum of POLR3-related leukodystrophy caused by *POLR1C* mutations [19,20,21,22]. The data reported here reinforced the association between *POLR1C* mutations and hypomyelinating leukodystrophy and emphasized the relevance of combining clinical features, characteristic MRI patterns, and multi-omics to reach a definitive diagnosis in undiagnosed cases presenting hypomyelination and ataxia.

## Figures and Tables

**Table 1:** Demographic and genetic characteristics of our patient and reported patients with *POLR1C* mutations.

Patient	Family	Sex (F/M)	Ethnicity	Consanguinity	Age at Last Assessment (Years)	Age of Onset (Years)	Genetic Characteristics	
							Mutation 1	Mutation 2
Index	XXIV	F	Caucasian (Spanish)	No	14	2.5	c.193A>G;	c.836G>A;
							p.Met65Val	p.Arg279Gln
1 [19]	I	M	Libyan	Yes	8	0.5	c.95A>T;	c.95A>T;
							p.Asn32Ile	p.Asn32Ile
2 [19]	II	M	Hungarian	No	10	1	c.221A>G;	c.221A>G;
							p.Asn74Ser	p.Asn74Ser
3 [19]	III	M	Asian (Chinese)	No	4	1	c.436T>C;	c.883_885delAAG;
							p.Cys146Arg	p.Lys295del
4 [19]	IV	F	Caucasian (Armenian/Russian)	No	6	2.5	c.77C>T;	c.326G>A;
							p.Thr26Ile	p.Arg109His
5 [19]	V	F	Caucasian	No	9	1.5	c.193A>G;	c.572G>A;
							p.Met65Val	p.Arg191Gln
6 [19]	VI	F	Caucasian (Turkish)	Suspected	18	4	c.326G>A;	c.970G>A;
							p.Arg109His	p.Glu324Lys
7 [19]	VII	M	Caucasian	No	33	2	c.395G>A;	c.461_462delAA;
							p.Gly132Asp	p.Lys154Argfs*4
8 [19]	VIII	F	Caucasian	No	2	1	c.281T>C;	c.785T>C;
							p.Val94Ala	p.Ile262Thr
9 [20]	IX	F	Tunisian	Yes	25	5	c.863T>C;	c.863T>C;
							p.Phe288Ser	p.Phe288Ser
10 [22]	X	M	Caucasian (British)	No	NA	0	c.69+1G>A;	c.836G>A;
							p.Asn24Asnfs55*; (prediction)	p.Arg279Gln
11 [22]	XI	M	Caucasian	No	NA	4	c.916_920delTATAT;	c.938C>T;
12 [22]		M					p.Tyr306Leufs*4	p.Thr313Met
13 [22]	XII	M	Caucasian (Dutch)	No	NA	2	c.193A>G;	c.733G>A;
							p.Met65Val	p.Val245Met
14 [22]	XIII	F	Caucasian (English)	No	NA	3	c.313A>T;	c.916_920delTATAT;
							p.Ile105Phe	p.Tyr306Leufs*4
15 [22]	XIV	F	Caucasian (English)	Yes	NA	2	c.836G>A;	c.836G>A;
							p.Arg279Gln	p.Arg279Gln
16 [22]	XV	F	Norwegian	No	NA	0.3	c.88C>T;	c.916_920delTATAT;
							p.Pro30Ser	p.Tyr306Leufs*4
17 [22]	XVI	F	Caucasian (English)	No	NA	0	c.221A>G;	c.502G>A;
							p.Asn74Ser	p.Val168Met + splicing error
18 [22]	XVII	F	Caucasian	No	NA	6	c.79A>G;	c.349G>C;
							p.Thr27Ala	p.Ala117Pro
19 [22]	XVIII	F	Caucasian	No	NA	0.4	c.322C>T;	c.325C>T;
							p.His108Tyr	p.Arg109Cys
20 [22]	XIX	F	African/American	No	NA	2	c.70-1G>A;	c.835C>T;
							p.Asn24Profs27* (prediction)	p.Arg279Trp
21 [22]	XX	F	Caucasian	No	NA	0	c.699C>G;	c.883_885delAAG;
							p.Tyr233*	p.Lys295del
22 [22]	XXI	M	Caucasian	No	NA	1	c.88C>T;	c.615delC;
23 [22]		F				0	p.Pro30Ser	p.Gln206Lysfs*48
24 [22]	XXII	F	Asian	No	NA	3.5	c.77C>T;	c.77C>T;
							p.Thr26Ile	p.Thr26Ile
25 [21]	XXIII	M	Asian (Korean)	NA	NA	5	c.698_699insAA;	c.713A>G;
26 [21]		F					p.Tyr233fs	p.Asp238Gly

F: female; M: male; NA: not available.

**Table 2:** Clinical characteristics of our patient and reported patients with *POLR1C* mutations.

Patient	Symptoms at Onset ^1^	Develop-mental Delay ^1^	Age at Walking without Support (Months) ^1^	Abnormal Cognition ^1^	Cerebellar Signs	Tremor ^1^	Pyramidal Signs	Dystonia	Myoclonus	Age at Wheelchair (Years)	Myopia	Dental AbN	Hypogonadotropic Hypogonadism
Index	Delayed motor development, tremor, ataxia, dysmetria	+	28	+	+	+	+	+	+	7	+	−	−
1 [19]	Delayed motor development	+	22	+	+	+	+	−	−	3	−	+	Too young
2 [19]	Ataxia, tremor, head titubation	+	18	+	+	+	+	−	−	9 *	−	−	Too young
3 [19]	Delayed motor development and failure to thrive	+	Never autonomously	+	+	+	+	+	−	Always	−	−	Too young
4 [19]	Tremor, dysmetria	+	15	−	+	+	+	−	−	− *	−	+	Too young
5 [19]	Delayed motor development	+	24	+	+	+	+	−	−	Always *	+	−	Too young
6 [19]	Clumsy gait, frequent falls	−	18	+	+	+	+	+	−	9 (long distances) *	+	−	−
7 [19]	Delayed motor development	+	Never autonomously	+	+	+	−	−	+	Puberty	+	−	−
8 [19]	Delayed motor development	+	24 (with support)	−	+	+	−	−	−	−	−	+	Too young
9 [20]	Tremor, ataxia	−	12	−	+	+	+	+	+	21 *	−	−	−
10 [22]	NA	NA	NA	NA	+	NA	+	−	NA	NA	−	+	Too young
11 [22]	NA	NA	NA	NA	+	NA	−	−	NA	−	−	+	−
12 [22]	NA	NA	NA	NA	+	NA	−	−	NA	−	+	+	−
13 [22]	NA	NA	NA	NA	+	NA	−	−	NA	−	+	−	−
14 [22]	NA	NA	NA	NA	+	NA	−	−	NA	12	+	+	Too young
15 [22]	NA	NA	NA	NA	+	NA	+	+	NA	7	−	−	Too young
16 [22]	NA	NA	NA	NA	+	NA	−	+	NA	0	+	+	Too young
17 [22]	NA	NA	NA	NA	+	NA	+	+	NA	0	+	+	Too young
18 [22]	NA	NA	NA	NA	+	NA	+	−	NA	−	−	+	−
19 [22]	NA	NA	NA	NA	+	NA	+	−	NA	0	−	+	−
20 [22]	NA	NA	NA	NA	+	NA	+	−	NA	11	−	−	Too young
21 [22]	NA	NA	NA	NA	+	NA	+	+	NA	3	+	+	Too young
22 [22]	NA	NA	NA	NA	+	NA	+	+	NA	4	+C	+	Too young
23 [22]	NA	NA	NA	NA	NA	NA	NA	NA	NA	NA	NA	−	Too young
24 [22]	NA	NA	NA	NA	+	NA	−	−	NA	−	+	+	Too young
25 [21]	Tremor, ataxia	+	NA	+	+	+	+	NA	−	NA	+	−	−
26 [21]	Tremor, ataxia	+	NA	+	+	+	+	NA	+	NA	+	−	−

AbN: abnormalities; +: present; −: absent; NA: not available; C: cataracts.^1^ General information available for patients 10 to 24 at Gauquelin et al., 2019. * Fast deterioration with infection.

**Table 3:** Main MRI characteristics of our patient and reported patients with *POLR1C* mutations.

Patient	Diffuse Hypomyelination	Cerebellar Atrophy	Thin Corpus Callosum
Index	+	+	+
1 [19]	+	−	+
2 [19]	+	−	+
3 [19]	+	−	+
4 [19]	+	+	+
5 [19]	+	+	+
6 [19]	+	+	+
7 [19]	+	+	+
8 [19]	+	+	+
9 [20]	+	+	+
10 [22]	+	−	+
11 [22]	+	+	+
12 [22]	+	+	+
13 [22]	+	+	+
14 [22]	+	+	+
15 [22]	+	+	+
16 [22]	+	−	−
17 [22]	+	+	+
18 [22]	+	+	+
19 [22]	+	+	+
20 [22]	+	+	+
21 [22]	+	−	+
22 [22]	+	+	+
23 [22]	NA	NA	NA
24 [22]	+	+	+
25 [21]	+	−	NA
26 [21]	+	−	NA

MRI: magnetic resonance imaging; +: present; −: absent; NA: not available.

## Data Availability

The data that support the findings of this study are available from the corresponding author upon reasonable request.

## Supplementary Materials

Supplementary Materials can be found at https://www.mdpi.com/1422-0067/22/6/2990/s1.
