# Supplementary material for: Combined Genome, Transcriptome and Metabolome Analysis in the Diagnosis of Childhood Cerebellar Ataxia
Source: Int J Mol Sci. 2021 Mar 15;22(6):2990. doi: 10.3390/ijms22062990 (PMC8002209; doi:10.3390/ijms22062990)
Supplement: Supplementary file 1 [file ijms-22-02990-s001.zip › Suppl material.docx]

Combined genome, transcriptome and metabolome analysis in the diagnosis of childhood cerebellar ataxia

Ana Ching-López ^1^, Luis Javier Martinez-Gonzalez ^2^, Luisa Arrabal ^3^, Jorge Saiz ^4^, Ángela Gavilán ^5^, Coral Barbas ^4^, Jose Antonio Lorente ^2,6^, Susana Roldán ^3^, Maria José Sánchez ^1,7,8,^*^,†^ and Purificacion Gutierrez-Ríos ^8,†^

***** Correspondence: mariajose.sanchez.easp@juntadeandalucia.es

^†^ These authors have contributed equally

**Supplementary Material**

**Methodology for the untargeted metabolomics analysis**

*Sample preparation procedure*

Plasma sample preparation for liquid chromatography–mass spectrometry (LC–MS) entailed the following steps: (1) thaw sample on ice, vortex for 2 min and transfer 100 µL of plasma into an Eppendorf tube; (2) for protein precipitation, add 300 µL of cold (-18˚C) methanol/ethanol (1:1 v/v), vortex for 1 min, incubate on ice for 5 min and vortex again briefly; (3) centrifuge at 13000 rpm, 4˚C, for 20 min; and (4) transfer the supernatant into the LC–MS system.

Plasma sample preparation for gas chromatography–mass spectrometry (GC–MS) entailed the following steps: (1) thaw sample on ice, vortex for 2 min and transfer 40 µL of plasma into an Eppendorf tube; (2) for protein precipitation, add 120 µL of cold acetonitrile, vortex for 2 min and incubate on ice for 5 min; (3) centrifuge at 15400 rpm, 4˚C, for 10 min; and (4) transfer the supernatant into a GC vial with insert; (5) evaporate in a Speedvac at 30°C until dry; (6) add 10 µL of O-methoxyamine hydrochloride (15 mg/mL) in pyridine and vortex for 5 min; (7) sonicate for 2 min and vortex for 2 min (repeat step a total of three times); (8) incubate at room temperature for 16 h in darkness for the reaction of the methoxymation; (9) add 10 µL of N,O-Bis(trimethylsilyl)trifluoroacetamide (BSTFA) with 1 % trimethylchlorosilane (TMCS) and incubate 1 h at 70 ˚C for the silylation; (10) add 100 µL of 10 ppm C18:0 methyl ester in n-heptane as internal standard, vortex for 2 min and centrifuge at 2500 rpm, 20˚C, for 15 min; and (11) transfer into the GC–MS system.

Plasma sample preparation for capillary electrophoresis–mass spectrometry (CE–MS) entailed the following steps: (1) thaw sample on ice, vortex for 1 min and transfer 100 µL of plasma into an Eppendorf tube already filled with 100 µL of 0.2 M formic acid with 5 % acetonitrile and 0.4 mM methionine sulfone as internal standard; (2) vortex for 2 min and transfer to a Millipore filter (30 kDa protein cutoff); (3) centrifuge at 2000 rpm, 4˚C, for 70 min; and (4) transfer the filtrate into a CE vial where the volume is directly injected into the CE–MS system.

Urine samples for LC–MS were thawed on ice and vortexed prior injection into the LC-MS system (without pre-treatment). For CE–MS, sample procedure entailed: (1) thaw on ice and vortex for 1 min and transfer 200 µL into an Eppendorf tube; (2) centrifuge at 13200 rpm, 4˚C, for 20 min; (3) transfer 100 µL of the supernatant into an Eppendorf tube already filled with 400 µL of 0.125 M formic acid with 0.25 mM methionine sulfone as internal standard; (4) vortex for 1 min and centrifuge at 13200 rpm, 4˚C, for 10 min; and (5) transfer 200 µL of the supernatant into a CE vial where the volume is directly injected into the CE–MS system.

Quality control (QC) samples were independently prepared for each technique and followed the same procedure as applied for experimental samples. QC samples were always injected at the beginning of each analytical run, followed by samples randomized independently. A QC sample was rerun after each block of 5 samples.

*LC–MS analysis*

Analyses were performed using a UHPLC system (Agilent 1290 Infinity LC System, Waldbronn, Germany) coupled to a LC-QTOF-MS (6520) analyzer (Agilent Technologies, CA, USA). The UHPLC system consisted of a degasser, a binary pump and an autosampler (maintained at 4 ˚C). For the plasma samples, 10 µL of each sample were injected into a reverse-phase Discovery® HS C18 (15 cm x 2.1 mm x 3 µm) (Supelco, PA, USA) maintained at 40 ˚C. The flow rate was 0.6 mL/min with solvent A composed of water with 0.1% formic acid, and solvent B composed of acetonitrile with 0.1% formic acid. The chromatographic gradient started at 25% of solvent B, increased to 95% in min 35, then decreased down to 25% from 35−36 min, and kept at 25% of solvent B from 36−45 min. For urine samples, 5 µL of each sample were injected into a reverse-phase Ascentis® Express C18 (5 cm x 2.1 mm x 1.7 µm) (Supelco, PA, USA) maintained at 30 ˚C. The flow rate was 0.5 mL/min with solvent A composed of water with 0.1% formic acid and, solvent B composed of acetonitrile with 0.1% formic acid. The chromatographic gradient started at 0% of solvent B, was kept for 0.5 min, was increased to 9% in min 2, 20% in min 5, 45% in min 8 and 100% in min 9.5. This condition was kept until min 11, decreased to 0% in min 11.5 and maintained between min 11.5 and min 20. Capillary voltage was set to 3000 V for both ionization modes; the drying gas flow rate was 10.5 L/min at 330 ˚C and gas nebulizer 52 psig; fragmentor voltage was 175 V for positive and negative ionization mode, skimmer voltage was set at 65 V and octopole radio frequency voltage (OCT RF Vpp) at 750 V. Data were collected in full scan mode, from 50 to 1000 m/z for positive ionization mode and from 50 to 1100 for negative mode, at a rate of 1.2 scan/s. Accurate mass measurements were obtained by means of an automated calibrant delivery system using a Dual Agilent Jet Stream Electrospray ionization (Dual AJS ESI) source that continuously introduces a calibrant solution containing reference masses at m/z 121.0509 and 922.0098 for positive and m/z 68.9957, 119.0363, 966.0007 for negative ionization mode. Data acquisition was performed with MassHunter Workstation Data software B.06.01 (Agilent Technologies, CA, USA).

*GC–MS analysis*

Analyses were performed using a 7890A from Agilent Technologies (Waldbronn, Germany) coupled to an inert mass spectrometer with triple-Axis detector 5975C (Agilent Technologies, CA, USA). The injection volume of derivatized samples was set at 2 µL using an autosampler 7693 (Agilent Technologies, CA, USA). Samples were automatically injected in split mode, with a 1:10 split ratio, into an Agilent deactivated glass wool split liner. Separation of the compounds was achieved using a 10m Agilent J&W pre-column integrated with a 122–5332G column: DB5-MS 30 m length, 0.25 mm i.d. and 0.25 m film consisting of 95% dimethyl/5% diphenyl polysiloxane (Agilent Technologies, CA, USA). Helium was used as the carrier gas with a constant flow rate of 1 mL/min. The lock of the retention time (RTL) relative to the internal standard (methyl stearate C18) peak at 19.66 min was performed. The chromatographic separation consisted of temperature gradient for the column oven programmed at 60 ˚C (maintained for 1min), then raised by 10 ˚C/min until it reached 325 ˚C, and held at that temperature for 10 min before cooling down. The temperatures of the injector and the transfer line were set at 250 ˚C and 280 ˚C, respectively. MS system: the electron impact ionization operating parameters were set as follows: filament source temperature, 230 ˚C; electron ionization energy, 70 eV. Mass spectra acquisition range was set at 50–600 m/z at a scan rate of 2 spectra/s. For retention index (RI) determination, a mixture of n-alkanes (C8-C40) and a mixture of FAMEs (C8:0-C22:1, n9) were run prior to the samples. Data were acquired using the Agilent MSD ChemStation software B.06.02 (Agilent Technologies CA, USA).

*CE–MS analysis*

Analyses were performed on a 7100 CE system (Agilent Technologies, CA, USA) coupled to a 6224 accurate mass TOF MS (Agilent Technologies CA, USA), equipped with an electrospray ionization (ESI) source. The separation was performed in a capillary with an inner diameter of 50 µm and a total length of 100 cm. Before each analysis, the capillary was conditioned with background electrolyte (BGE) (0.8 M formic acid solution in 10% methanol (v/v)) for 5 min at 950 mbar. Sample injections were performed for 50 s at 50 mbar. After the injection of each sample, a plug of BGE was injected at 100 mbar pressure during 20 s to improve repeatability. Separation was performed at 30 kV with a constant pressure of 25 mbar applied from the inlet at 20 ˚C. Analysis time was 40 min. Data were acquired in positive, full scan MS mode, within the range 74–1000 m/z at a rate of 1.0 scan/s. Other parameters for the MS were: fragmentor 125 V, skimmer 65 V, octopole 750 V, drying gas temperature 200 ˚C, flow rate 10 L/min and capillary voltage 3500 V. The sheath liquid used for the detection consisted of 50% methanol, 50% ultrapure water, 1 mM formic acid and two reference masses (purine: m/z 121.0509 and HO0921: m/z 922.0098) at a flow rate of 0.6 mL/min (1:100 split). Data acquisition was performed with ChemStation software B.06.01 (Agilent Technologies Santa Clara, CA, USA) and MassHunter Workstation Data software B.06.01 (Agilent Technologies, CA, USA) controlled the MS performance.

*Data treatment*

All data obtained after LC–MS or CE–MS analysis were treated in the same way. Before reprocessing, an initial qualitative analysis was performed to check all the electropherograms/chromatograms in order to detect possible outliers due to instrumental errors. Raw data acquired were processed to provide structured data in an appropriate format for data analysis. Using the Batch Molecular Feature Extraction (MFE) tool in MassHunter Profinder software B.08.00 (Agilent Technologies CA, USA), data collected by CE–MS and LC–MS were cleaned from background noise and unrelated ions. The algorithm of the MFE tool groups ions by isotopic distribution, charge-state envelope and/or presence of adducts and/or loss of neutral molecule and dimers to generate a list of all the possible components (features). The possibility of neutral loss of water was also included in each case. Each compound was described by mass, migration time (MT) (CE–MS) or retention time (RT) (LC–MS), and abundance. Data were filtered based on their quality assurance, whereby variables were retained if they were present in at least 80% of the QCs or absent in QCs, present in 80% of one of the two groups and present with a QC relative standard deviation (RSD) below 30%. For the data obtained in GC–MS, the total ion chromatograms (TICs) obtained after the analysis were inspected based on both quality of the chromatograms and internal standard signals. The files were exported to Agilent MassHunter Unknowns Analysis software version B.08.00 for the deconvolution process and the metabolite identification of raw data collected by GC–MS analysis. Agilent MassHunter Quantitative Analysis software version B.08.00 was used for the assignment of the target and qualifiers ions and peak area integration. Prior to the statistical analysis, sample areas were normalized by the internal standard abundance in order to minimize the response variability coming from the instrument. Data were filtered by the coefficient of signal variation (CV) in QCs, considering as acceptable values lower than 30%.

The features or metabolites from the two samples were compared by their absolutes differences in abundance, and that was expressed as the logarithm base 2 of the fold change (log2FC). Those features or metabolites having a log2FC ≥ 1.5 were considered significant and were selected.

*Metabolite identification*

Compound identification from LC–MS and CE–MS data that were found to be significant in class separation was performed by matching each accurate m/z measurements of detected chromatographic peaks to leading online MS databases as METLIN (http://metlin.scripps.edu), KEGG (https://www.genome.jp/kegg/genome.html), HMDB (http://hmdb.ca) and LIPID MAPS (http://www.lipidmaps.org/) using the CEU Mass Mediator tool (http://ceumass.eps.uspceu.es). The identification of m/z was performed considering each of the possible adducts as described for MFE. For CE–MS data, compound identification was confirmed by comparing masses and relative migration times to those of an in-house standards library. For LC–MS data, the samples containing higher concentrations of the compounds of interest were re-analyzed in MS/MS mode selecting the precursor ion according to the MFE process and recording all the product ions produced at different collision energies. At least five of the most abundant product ions were selected, when possible, for the search in the mentioned online databases. Whenever the databases did not show any match, the different possibilities of fragmentation were studied considering the precursor ion and the product ion for the identification of the metabolites. For GC–MS data, compound identification was performed with the Fiehn library version 2008 and the CEMBIO in-house spectral library, and always by comparing both retention time (RT) and spectra extracted during the deconvolution against each compound included in the library. For the non-identified compounds, a mixture of n-alkanes that was analyzed at the beginning of the analytical run was used as reference for RT/RI comparison with a commercial mass spectra library —NIST (National Institute of Standards and Technology) version 2.2 2014. Those metabolites with a spectrum score higher than 80% and concordant RI based on the n-alkane scale were putatively identified according to NIST.
